# Retraction: ISL1 protein transduction promotes cardiomyocyte differentiation from human embryonic stem cells

**DOI:** 10.1371/journal.pone.0352117

**Published:** 2026-06-25

**Authors:** The PLOS One Editors

After this article [1] was published, concerns were raised with Figs 1, 2 and S1. Specifically,

- In the *Brachyury*, *Actinin*, *Mef2c*, *Gata4*, *MHC*, and *cTnT* panels of Fig 1, the error bars appear more uniform than would be expected across all time points.
- In Fig 2C, there appear to be vertical discontinuities between the Dilution Buffer negative control lane and the 5 μg lane, and between the 10 µg and 50 µg lanes.
- In Fig 2E, there appears to be a vertical discontinuity between the 2h and 6h lanes of the Cell lysate of vehicle group panel.
- In Fig S1B, multiple bands in different gene rows and/or at different time points appear more similar to one another than would be expected, including:
  - *BRACHYURY* D4 and *MEF2C* D7
  - *BRACHYURY* D5 and *BRACHYURY* D8
  - *BRACHYURY* D7 and *ISL1* D5
  - *ISL1* D3 and *GATA4* D8
  - *ISL1* D7, *GAPDH* D1 and *GAPDH* D8
  - *MEF2C* D9 and *GAPDH* D12
  - *MEF2C* D10 and *GATA4* D10
  - *βMHC* D11, *βMHC* D13 and *MLC2v* D10
  - There appear to be multiple vertical discontinuities across all panels in Fig S1B.

The first author stated that with regard to the qRT-PCR graphs in Fig 1, they acknowledge the appearance of uniform error bars and believe this reflects issues with graph preparation or spreadsheet formatting. They stated that the data underlying Fig 1 is no longer available, with the exception of the data for *ISL1*, *NKX2–5* and *TBX5* and partial data for *Brachyury*, *Mef2c*, *cTnT*, *Gata4* and *MHC*. In the absence of the full underlying data for all panels of concern in Fig 1, these concerns cannot be fully resolved.

With regards to the concerns with Fig 2C, the first author stated the experimental samples shown originated from the same underlying blot, and the observed discontinuities reflect figure assembly. They also stated that their standard experimental practice at the time of publication of [1] was to leave an empty lane between negative controls and experimental samples to minimize the possibility of cross-contamination. For Fig 2E, the first author stated that the 2h time point was run on the same blot as the rISL1-treated samples, and that as both β-actin and ISL1 were probed simultaneously, normalization between samples was still possible. They also stated that during figure preparation, the vehicle 2h lane was repositioned into the vehicle group panel. The underlying blots provided for Figs 2C and 2E do not appear to match the published panels and therefore the concerns with Figs 2C and 2E remain unresolved.

With regards to the concerns with Fig S1B, the first author stated that the complete original uncropped gel files for Fig S1B are no longer available. The concerns with Fig S1B therefore cannot be fully resolved.

In light of the above concerns which call into question the reliability and integrity of the results in [1], the *PLOS One* Editors retract this article.

HF responded but expressed neither agreement nor disagreement with the editorial decision. MY, FF, ZG, HR, MA, BAM, HB, GHS, and NA either did not respond directly or could not be reached.
